# Detecting Depression in Patients with Coronary Heart Disease: a Diagnostic Evaluation of the PHQ-9 and HADS-D in Primary Care, Findings From the UPBEAT-UK Study

**DOI:** 10.1371/journal.pone.0078493

**Published:** 2013-10-10

**Authors:** Mark Haddad, Paul Walters, Rachel Phillips, Jacqueline Tsakok, Paul Williams, Anthony Mann, André Tylee

**Affiliations:** 1 School of Health Sciences, City University London, London, United Kingdom; 2 Health Services and Population Research Department, Institute of Psychiatry at King’s College London, London, United Kingdom; Chiba University Graduate School of Medicine, Japan

## Abstract

**Objective:**

People with coronary heart disease (CHD) are at heightened risk of depression, and this co-occurrence of conditions is associated with poorer outcomes including raised mortality. This study compares the diagnostic accuracy of two depression case finding instruments in CHD patients relative to a diagnostic standard, the revised Clinical Interview Schedule (CIS-R).

**Methods:**

The Patient Health Questionnaire (PHQ-9), the Hospital Anxiety and Depression Scale depression subscale (HADS-D) and the CIS-R depression module were administered to 803 patients identified from the CHD registers of GP practices in Greater London.

**Results:**

Of 730 recruited patients without previously identified depression, 32 (4.4%) met ICD-10 depressive episode criteria according to the CIS-R. For the PHQ-9 and HADS-D lower cut-points than those routinely recommended were associated with improved case identifying properties. The PHQ-9 appeared the superior instrument using a cut-point of ≥8 (sensitivity=94%; specificity=84%). Using categorical scoring the PHQ-9 was 59% sensitive and 95% specific. For the HADS-D using cut-point ≥5, sensitivity was 81% and specificity was 77%.

Areas under the curves (AUC) (standard error) were 0.95 (0.01) and 0.88 (0.02) for the PHQ-9 and HADS-D, and 0.91 (0.02) for PHQ-9 using the categorical algorithm. Statistically significant differences between AUCs of the PHQ-9 and the HADS-D favoured the former. Severity ratings compared across measures indicated inconsistency between recommended bandings: the PHQ-9 categorised a larger proportion of participants with mild and moderate depression.

**Conclusion:**

This is the first large-scale investigation of the accuracy of these commonly used measures within a primary care CHD population. Our results suggest that although both scales have acceptable abilities and can be used as case identification instruments for depression in patients with CHD, the PHQ-9 appeared diagnostically superior. Importantly, optimal cut-off points for depression identification in this population appear to differ from standard values, and severity ratings differ between these measures.

## Introduction

Depression is one of the commonest mental disorders, with a 12-month community prevalence of 4% to 7 % [1,2]. It is currently the third leading cause of burden of disease burden in the world, and the leading cause in middle- and high-income countries [3]. 

The risk of depression is significantly increased among people with chronic illnesses such as coronary heart disease (CHD), chronic obstructive pulmonary disease (COPD), diabetes, and asthma, with rates consistently found to be two- to three-times times higher than in the general population [4,5]. When depression co-occurs with a medical illness it is associated with poorer physical, mental, and social functioning in all age groups than either depression or physical illness alone [6]. In the case of CHD, co-occurrence with depression predicts a doubling of the risk of cardiac events in the years following myocardial infarction [7], and a similar increase in mortality [8].

Recognition of depression is important for treatment and monitoring, but a substantial body of research has found this to be poor in primary care and medical settings [9]. Difficulties in case identification appear to be amplified by the presence of concurrent medical conditions such as CHD [10,11]. In response to this, the use of validated brief self-report scales to improve case identification for depression has received much attention and been advocated by clinical guidelines in the USA [12], whilst in the UK, the Quality and Outcomes Framework (QOF) has since 2006 provided incentives for general practitioners’ (GPs) systematic use of a brief depression case identification instrument in particular vulnerable groups. 

Despite the relative ease of application and apparent utility of depression screening tools, there remain important questions about the effect of this approach for clinical outcomes in primary care patients [13]. A key problem with depression screening within an unselected primary care population is that the positive predictive value derived from available instruments is too low – primarily because of the relatively low prevalence of undisclosed depression in this patient group. However, the use of case-finding tools in high-risk groups is likely to yield a lower frequency of false-positive results and hence appears a more appropriate strategy than routine screening, and this is the basis of recommendations for systematic case identification among people at increased risk of depression because of past history or specific conditions such as CHD [7,14]. The American Heart Association, endorsed by the American Psychiatry Association, stress that the opportunity to screen for and treat depression in cardiac patients should not be missed, and recommend routine screening for depression in patients with CHD in the various settings where they receive care [7]. The UK National Institute for Health and Care Effectiveness, whilst not recommending routine depression screening in primary care [15], does advocate the continuing use of a two question depression screen [16] in the case identification of depression among people with patient with a chronic physical health problem [14]; and, alongside further psychosocial assessment of patients who respond positively, to consider using a validated measure to inform and evaluate treatment. Hence, use of depression case identifying measures (aside from a two-item screen) in the UK is largely for purposes of confirmation of diagnostic suspicion, indication of condition severity, and monitoring responses to management, rather than screening.

There remain important questions about the clinical value of depression screening: leading commentators note that there is a lack of evidence for this approach leading to improved outcomes, and voice associated concerns about its cost-effectiveness [17,18]. There may also be the potential for harms such as incorrectly identifying (false-positive results) and treating individuals for depression, providing treatment of symptoms that may be likely to be self-limiting, and the possible adverse psychological and behavioural consequences of identifying depression among individuals previously unconcerned about their mental health [19]. Alongside these key issues, there are uncertainties about the most appropriate instruments and scale cut-off points for use with CHD patients, whether for their use (as recommended in the USA) in screening, or for the purposes advocated in the UK of case identification and diagnostic confirmation. 

The Patient Health Questionnaire (PHQ-9) and the depression subscale of the Hospital Anxiety and Depression Scale (HADS-D) are commonly used to improve the identification and management of comorbid depression in people with medical conditions. Previous research has examined the validity and psychometric properties of these instruments among primary care patients [20], among patients with comorbid chronic conditions including CHD [21], and among recently discharged CHD patients [22], as well as those with a longer history of CHD [23]. A systematic review [24] of the diagnostic accuracy of these and other widely-used case identification tools for depression among patients with long-term medical conditions found that although most scales performed adequately using standard cut-points, the PHQ-9 (sensitivity=84%; specificity=88%) achieved greater overall accuracy than the HADS-D (sensitivity=75%; specificity=81%). 

A systematic review focussing on the adequacy of screening methods specifically for patients with cardiovascular disease [25] identified inconsistencies in the performance and optimal thresholds of these instruments between samples. Among the reviewed studies, a large-scale community study of CHD outpatients [23] found the standard PHQ-9 cut-point (≥10) had only 54% sensitivity and 90% specificity, with further post hoc analysis [26] indicating that a PHQ-9 cut-off score of ≥6 was optimal: 83% sensitive and 76% specific. This same PHQ-9 cut-off score of ≥6 was also indicated in a smaller study examining patients within 3 months of discharge following cardiac events [22] (sensitivity=83%; specificity=79%). Evaluations of the HADS-D in this patient group have similarly indicated a lowering of cut-points for optimal accuracy: thresholds of ≥6 (sensitivity=80%, specificity=82%) [22] or ≥4 for major depression (sensitivity=85%, specificity=75%) [27] have been advocated. 

Importantly, studies of the diagnostic accuracy of these instruments in CHD patients have recruited samples almost exclusively from either hospitalized patients or recently discharged patients with recent acute coronary syndrome or coronary revascularization; only one study of community based cardiology outpatients approximates to a primary care sample [26]. The lack of investigation of the characteristics and accuracy of depression case finding tools among a primary care CHD population is a limitation within this literature. This is important because primary care is the setting where there is most opportunity and potential value in identifying depression among people with CHD. 

A further problem evident in the literature is that although there are a large number of primary studies reporting evaluations of the characteristics of depression case identification tools, the overwhelming majority of these diagnostic accuracy studies fail to exclude patients who already have a diagnosis of or are receiving treatment for depression. A recent systematic review [28] identified that only around 5% of nearly 200 unique publications specifically removed depressed patients from the sample in which the tool was evaluated. Because screening is designed to identify those patients who may have a condition, but are neither seeking treatment nor have had the target condition otherwise recognised, then including patients already identified as cases does not represent the population among which this procedure would be used in real practice (termed *spectrum bias*). Failing to omit such patients compromises the validity of findings: the increased prevalence and severity of depression in the sample being examined may inflate the reported sensitivity of the index test in relation to the reference standard, and give rise to inaccurate and misleading estimates of the positive predictive value and new case yield that may be derived from the use of the test in normal clinical practice. 

As well as concerns about the accuracy of previous study findings and their generalizability to primary care CHD populations and uncertainties about the optimal scale thresholds for this patient group, previous studies indicate that the PHQ-9 and HADS-D differ in the proportion of people classified with mild, moderate, or severe depression [20,29], and it seems likely that these inconsistencies of categorisation may also be evident for people with CHD. Accordingly, this study aims to determine the performance characteristics of these two widely used depression scales in a large representative primary care based UK sample of patients with CHD from which those patients who already have a diagnosis of or are receiving treatment for depression have been excluded, and to examine the relative severity classifications derived from these instruments. 

## Methods

### Ethics statement

All study participants provided written informed consent and relevant documentation was retained in patients’ medical notes and the research records. This study including the consent procedure received ethical approval from the Bexley and Greenwich Research Ethics Committee (REC Reference: 07/H0809/38).

### Study Design

This study uses a cross-sectional design to compare the psychometric characteristics of the PHQ-9 and HADS-D, with reference to the CIS-R as a diagnostic standard. This study forms part of a wider exploration of depression and CHD in primary care in which these and other measures are used to examine prevalence, incidence, course, predictors, management, and experience of these commonly comorbid conditions [30]. 

### Setting

Patients were recruited from the CHD registers kept by general practices in South London (In 2004, the Quality & Outcomes Framework, part of the General Medical Services contract for England, introduced and incentivised arrangements for practices to identify their patient population with registers of particular clinical conditions to enable effective call and recall of patients in any disease category and in order to be able to report on relevant indicators). All patients on the CHD registers in 16 participating general practices were invited to participate in the study. Practices were selected with the intention of yielding participants with a mix of socio-economic and urban/rural status. 

### Participants

#### Inclusion criteria

Patients were eligible for study inclusion if they were aged 18 years and had been placed on the practice register of patients with coronary heart disease. The coronary heart disease register includes all patients in practices who have a past history of myocardial infarction, diagnosed angina, or had coronary artery revascularisation procedures such as coronary artery bypass grafting (CABG). Patients are assigned to the register on the basis of disease, investigation and intervention procedure codes within their electronic medical record.

#### Exclusion criteria

Patients were excluded if they did not have the necessary spoken or written language skills or were registered at the participating general practice as temporary patients. For this diagnostic accuracy study (though not for other studies within the UPBEAT-UK programme), patients already recognised or treated for depression were also excluded.

### Depression Severity Measures

The depression severity measures to be assessed comprised the HADS and PHQ-9, with accuracy compared to the CIS-R.

The PHQ-9 is a self-rating instrument for depression developed in the late 1990s from the Primary Care Evaluation of Mental Disorders (PRIME-MD) [31]. It consists of nine items designed to correspond to the Diagnostic and Statistical Manual of Mental Disorder (DSM-IV) [32]diagnostic criteria for major depressive disorder. Respondents rate the scale items from 0 to 3 according to the frequency of their experience over the previous 2-week period (not at all, several days, more than half the days, or nearly every day). As has been noted, a cut-off score equal or greater than 10 is most commonly used for depression identification, and a systematic review of studies in general practice conducted for most recent UK depression clinical guidelines [15] indicated a sensitivity of 82% and a specificity of 83% using this cut-point. Similar findings are reported from studies of people with chronic physical health problems or within general medical settings [24]. The PHQ-9 score can be used to indicate depression severity, however studies have found discrepancies in PHQ-9 severity bandings compared to other measures [20,29]. In addition to its use as a self-report case identification and severity measure, the PHQ-9 also includes a scoring algorithm that applies DSM-IV disorder criteria to the nine items wherein major depression is diagnosed if 5 or more of the 9 depressive symptom criteria have been present at least “more than half the days” over the past two weeks, and one of these symptoms is depressed mood or anhedonia. One of the 9 symptom criteria (“thoughts that you would be better off dead or of hurting yourself in some way”) counts if present at all, regardless of duration. If 2, 3, or 4 depressive symptoms have been present at least “more than half the days”, and one of the symptoms is depressed mood or anhedonia ‘other depressive syndrome’ may be diagnosed [33]. 

The HADS [34] rating scale is comprised of fourteen items, seven of which are designed to measure anxiety (HADS-A), and seven depression (HADS-D). Each of the items is scored on a four-point scale from zero (not present) to three (considerable). The item scores are summed, to provide sub-scale scores on the HADS-D and the HADS-A which may range between zero to 21. Studies most commonly employ a cut-point of ≥ 8 (eight and above) for each of the constituent subscales, as suggested by its authors, to indicate probable caseness. Studies of common mental disorders in primary care settings have utilised this cut-off score for caseness [35] and a systematic review reported that this threshold provides an optimal balance between sensitivity and specificity for both HADS-A and HADS-D [36]. The HADS-D has been used as for depression case-finding in a diverse and broad range of clinical groups including those with coronary heart disease [37], chronic fatigue, cancer, musculo-skeletal disease, as well as general population samples and primary care samples [38]. 

The CIS-R is a short lay interviewer administered structured interview schedule covering non-psychotic symptoms particularly those associated with depression and anxiety in the past month and past week [39]. It elicits responses to 14 areas of symptoms including depression, anxiety, panic, sleep and fatigue. Ratings can be summed to generate a total score, as well used to provide diagnostic categories according to ICD-10. Algorithms based on the type and number of reported symptoms enable catgorisation of depression episode severity as mild, moderate, or severe. The CIS-R is widely used, especially in the UK where it has been the main identification measure for common mental disorders in the national Psychiatric Morbidity Surveys carried out in 1993, 2000 and 2007. It has been found to have moderate validity with respect to the schedule for clinical assessment in neuropsychiatry (SCAN) [40]. In this study, the respondents’ answers to the CIS-R were used to define ICD-10 diagnoses of depressive episode (including mild, moderate and severe).

### Procedure

Sixteen practices in South East and South West London were recruited to participate in the study. The total practice population was 142,648 patients; 2.1% (2938/142,648) were on the QOF CHD registers. Thirty two per cent of people invited by their GP to participate in the study agreed to contact from the research team; of these 87.6% (803/917) consented to involvement and were recruited, representing 28.2% (803/2843) of those registered on the CHD registers of participating practices. Those patients who consented to participate were contacted by a researcher and the interview schedule including the HADS-D, the PHQ-9 and the CIS-R were administered face to face at their homes or GP practice. The PHQ-9 and HADS-D were delivered blind to the results of the diagnostic interview as the diagnostic categories were not calculated until the analysis stage. Further details of the method have been published elsewhere [30].

### Statistical analyses

All data were analysed using STATA 11.2. For all calculations, α was 0.05 and tests were two-tailed. A concurrent analysis was made of PHQ-9 and HADS-D scales. Convergent validity was assessed by Pearson product moment correlation between the two scales, and internal consistency of the scales was assessed using Cronbach’s alpha and item-total correlations.

Criterion validity was evaluated by determining the sensitivity and specificity for various cut-off scores on the PHQ-9 and HADS and for the PHQ-9 categorical algorithm in comparison to depressive episode as determined by CIS-R items (the criterion standard). We also plotted Receiver Operating Characteristic (ROC) curves for the scales (the true positive rate, sensitivity, plotted against the false positive rate, 100-Specificity) for all scale points to identify diagnostic superiority. Each point on the ROC plot represents a sensitivity/ specificity pair corresponding to a particular decision threshold. A test with perfect discrimination (no overlap in the two distributions) has a ROC plot that passes through the upper left corner (100% sensitivity, 100% specificity). Therefore the closer the ROC plot is to the upper left corner, the higher the test’s overall accuracy. A global test was used for statistical comparison of area under the curve (AUC). The AUCs were then compared pairwise to assess diagnostic superiority. We used the Youden index (sensitivity+specificity−1) to provide a single numerical estimation of the overall diagnostic effectiveness and to summarise the accuracy of the test instrument. This provides a value that may range between 0 and 1, where 1 means that the test is perfect. 

As other researchers have advocated for depression screening, particularly when a two-stage procedure may be used with initial identification followed-up with more detailed assessment [21,22], cut-off scores demonstrating maximal sensitivity and specificity of ≥75% were examined.

Convergence of the scales’ severity bandings was also compared relative to the CIS-R. 

## Results

### Sample Characteristics

The cohort consisted of 803 patients, of which 65 (8.1%) were currently diagnosed or being treated for depression. The findings reported here include only those recruited patients not recognised or treated for depression for whom all items of the PHQ-9, HADS-D and cis-R-D were completed (n=730). The sample was predominantly male (n=516; 71%), white (n=635, 87%), with a mean age of 71.2 years (S.D. = 10.5). Socio-economic status was represented using the index of multiple deprivation (IMD) scores for which higher scores indicate greater deprivation in the area. The median IMD was 18.4, with individual scores ranged from 1.7 to 61.6 (interquartile range 25.8). Patients in the cohort has been diagnosed with CHD for a mean duration of 10.4 years (S.D. = 7.9), and 80% were diagnosed with other comorbid medical conditions, most commonly hypertension (55%) and diabetes (25%); two or more comorbid medical conditions were recorded for 48% of patients. Current chest pain was present in 44% of the sample population, and 42% had documented history of myocardial infarction.

### Depression Measures

Of the recruited patients without previously identified or treated depression, 32 (4.4%) met the diagnostic criteria for ICD-10 depressive episode according to the CIS-R, (53 [6.6%] when analysis included the entire cohort), and of these 11 (17 without exclusion of patients already recognised as depressed) were identified as severely depressed. Mean scores on the PHQ-9 and HADS-D were 4.5 (S.D. =5.4, range 0-27) and 3.1 (S.D. =3.5, range 0-19), respectively. 

78 patients, 10.7%, were identified as depressed by HADS-D ≥8 (103 patients [13%] among the entire cohort), whilst 100 patients, 13.6%, scored ≥10 on the PHQ-9 (136 patients [17%] when the analysis was unrestricted). The demographic and depression status characteristics of the sample are shown in Table 1. 

**Table 1:** Sample characteristics of the primary care coronary heart disease register patients.

		No depressive disorder (CIS-R) (n=698)	Depressive disorder (CIS-R) (n=32)	P value
Female		201 (28.8%)	13 (40.6%)	0.151
Age, years (mean, SD)		71.44 (10.44)	65.28 (10.81)	0.001
Married		454 (65.0%)	17 (54.8%)	0.245
Employed		128 (18.4%)	10 (32.3%)	0.053
Current chest pain		287 (41.1%)	27 (84.4%)	<0.001
IMD score (mean, SD)		19.75 (13.96)	24.16 (13.25)	0.0803
PHQ-9 score (mean, SD)		3.60 (4.23)	14.84 (5.32)	<0.001
PHQ categorical algorithm	Other depression	82 (11.6%)	11 (34.4%)	<0.001
	Major depression	33 (4.7%)	19 (59.4%)	
HADS-D score (mean, SD)		2.84 (2.99)	8.06 (3.44)	<0.001

#### Internal consistency, homogeneity and convergent validity

The internal consistency (measured within the entire cohort) of both depression scales was high: Cronbach’s α for the PHQ-9 was 0.85 (n=801) and for the HADS (D), 0.80 (n=799). This indicates that both scales exhibit acceptable internal consistency with little likelihood of item redundancy.

Item total correlations were consistently satisfactory for each item of each questionnaire: the Pearson Product moment correlations ranged from 0.21 to 0.61 for the PHQ-9 and from 0.26 to 0.57 for the HADS-D. These values are all above the threshold 0.2 and therefore indicate that all individual items correlate with the scales from which they originate.

Assessment of convergent validity included participants who had completed both questionnaires completely (n=797). The Pearson’s correlation coefficient between HADS-D and PHQ-9 total scores was substantial r=0.71. For HADS-D and PHQ-9, r^2^=0.64, that is HADS-D sum score accounted for 64% of the variance in the PHQ-9 scores, providing good evidence of the extent to which the scales measure the same construct.

#### Criterion Validity

The operating characteristics of the scales are shown in Table 2, calculated for the 730 participants completing all instrument items and not previously identified as depressed. At least three potential cut-off points are shown for each instrument, calculated using HADS-D sum scores, PHQ-9 sum scores and the PHQ-9 categorical algorithm. Additional tables provided as Supporting Information (Tables S1-S3) show the results for all cut-off scores for both instruments. 

**Table 2:** Depressive disorder: PHQ-9 algorithm, PHQ-9 and HADS (D) operating characteristics.

	Sensitivity (95% CI)	Specificity (95% CI)	Positive Likelihood Ratio	Negative Likelihood ratio	Youden Index	Positive Predictive Value (%)	Negative Predictive Value (%)
**PHQ (n=730)**							
Cut-off point ≥7	93.8 (79.2, 99.2)	80.7 (77.5, 83.5)	4.8	0.1	0.74	18.2	99.7
Cut-off point ≥8	93.8 (79.2, 99.2)	83.8 (80.9, 86.5)	5.8	0.1	0.78	21.0	99.7
Cut-off point ≥9	87.5 (71.0, 96.5)	86.7 (83.9, 89.1)	6.6	0.1	0.74	23.1	99.3
Cut-off point ≥10	84.4 (67.2, 94.7)	89.8 (87.3, 92.0)	8.3	0.2	0.74	27.6	99.2
**PHQ-9 Categorical algorithm (n=730)**							
Major depression	59.4 (40.6, 76.3)	95.3 (93.4, 96.7)	12.6	0.4	0.547	36.5	98.1
Other depression	93.8 (79.2, 99.2)	83.5 (80.6, 86.2)	5.7	0.1	0.773	20.7	99.7
**HADS(n=730)**							
Cut-off point ≥3	100.0 (79.2, 99.2)	57.0 (53.3, 60.7)	2.3	0.0	0.57	9.6	100.0
Cut-off point ≥4	93.8 (63.6, 92.8)	67.8 (64.2, 71.2)	2.9	0.1	0.62	11.8	99.6
Cut-off point ≥5	81.3 (46.8, 81.4)	76.7 (73.3, 79.7)	3.5	0.2	0.58	13.8	98.9
Cut-off point ≥6	65.6 (40.6, 76.3)	83.1 (80.1, 85.8)	3.9	0.4	0.49	15.1	98.1
Cut-off point ≥7	59.4 (34.7, 70.9)	88.3 (85.6, 90.5)	5.1	0.5	0.48	18.8	97.9
Cut-off point ≥8	53.1 (26.4, 62.3)	91.4 (89.1, 93.4)	6.2	0.5	0.45	22.1	97.7

Overall, the instruments had sensitivities of 53% to 94% and specificities of 57% to 91% using standard cut-points and those suggested by prior studies (Table 2). 

Of the evaluated cut-points, PHQ-9 at ≥8 appeared to provide the optimal test characteristics in this population: although a similar proportion of cases of depression was identified at a lower cut-point, the combination of test values (sensitivity=94%; specificity=84%; Youden Index 0.78) supports the selection of this cut-point. The difference in performance between the cut-point routinely recommended (≥10) and that identified in this analysis was relatively modest for the PHQ-9; whilst for the HADS-D the standard cut-point of ≥8 provided a low sensitivity, 53%, with adequate specificity of 91%. The HADS-D at ≥5 demonstrated a sensitivity and specificity values of 81% and 77% respectively, whilst for a lower cut-point specificity fell below 75%.

PHQ-9 scoring by the categorical algorithm for major depression provided sensitivity and specificity values of 59% and 95%; whilst for ‘other depression’ respective values were 94% and 84%. 

**Figure 1:**
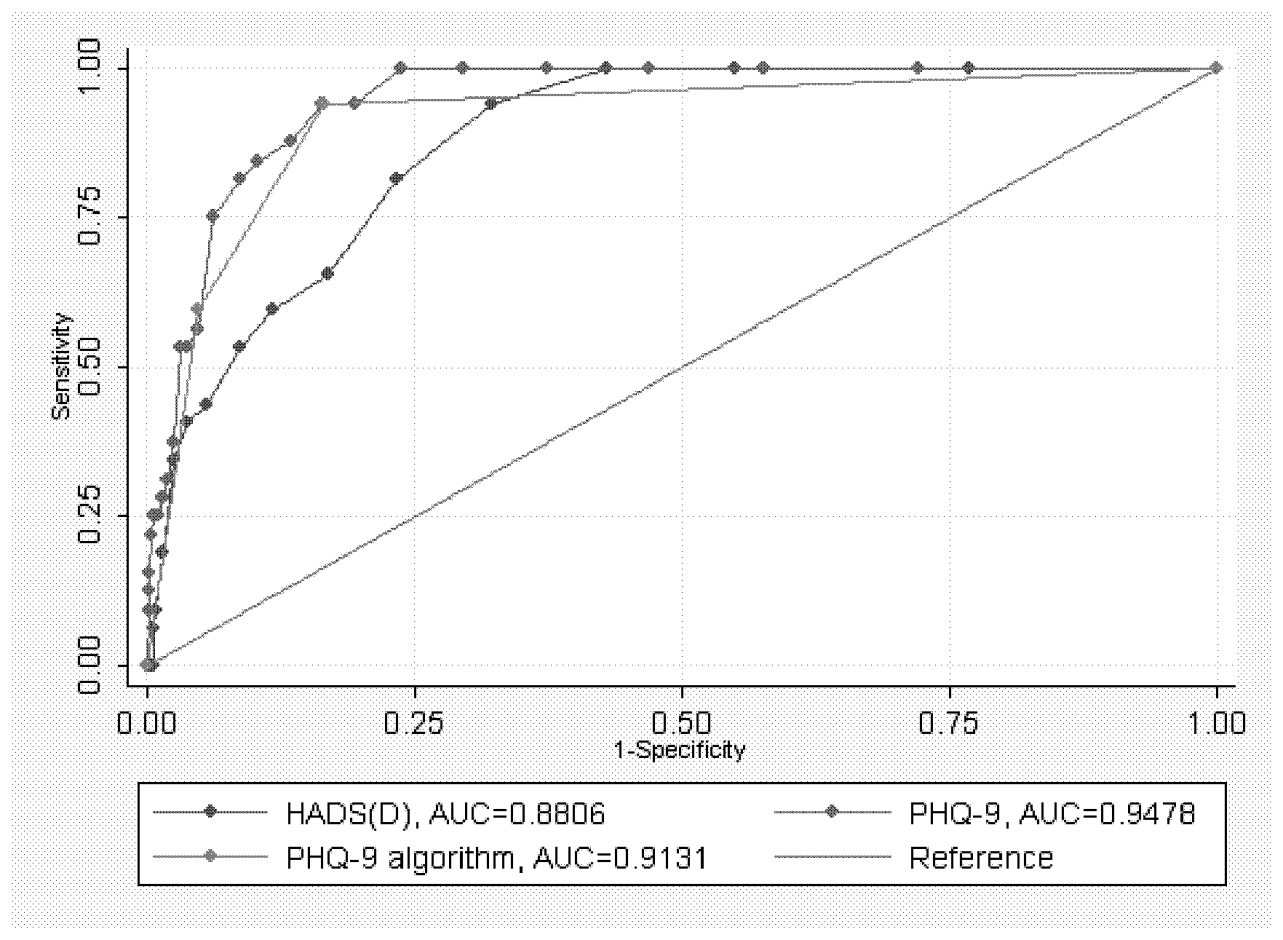
Receiver operating characteristic curves for PHQ-9, HADS-D, and PHQ-9 categorical algorithm; CIS-R was the criterion standard.

The AUCs of the PHQ-9, the PHQ-9 algorithm and the HADS-D for detecting depressive episode were 0.95 (SE 0.01, 95% CI 0.92 - 0.97), 0.91 (SE 0.02, 95% CI 0.86 - 0.96) and 0.88 (SE 0.02, 95% CI 0.84 - 0.92) respectively (Figure 1). Statistical comparison using the global test for the AUCs of the PHQ algorithm, the PHQ and HADS-D over all possible cut-off points was statistically significant (P<0.01). Pairwise statistical comparison of the AUCs showed that differences between the PHQ-9 and HADS-D sum scores (P<0.01) and between the PHQ score and PHQ algorithm (P=0.03), were statistically significant, suggesting the PHQ-9 is diagnostically superior to the other approaches for identifying depression episode. 

Pairwise statistical comparison between the HADS and the algorithm did not show a significant difference (P=0.18), suggesting that neither is diagnostically superior to the other in identifying depression.

PPV values for all the instruments tended to be low (10% to 28%), whilst the NPV values were very high (98% to 100%), implying that there is a high probability that depression is absent when the PHQ-9 test is negative, but that risk of false positives is high when these measures are used with a primary care CHD population. 

The PHQ-9, using summed scoring and a cut-point of ≥8 appeared the best performing instrument for use with this patient group. 

Diagnostic accuracy analyses were also conducted without excluding those patients already recognised or treated for depression: the same PHQ-9 cut-point (≥8) was found to be optimal, with similar sensitivity (94%) and specificity values (82%), however (because of the higher prevalence), the PPV value was 27% rather than 21%. 

#### Severity bandings

As noted, CIS-R algorithms enable ICD-10 depressive episode to be categorised by severity, and similarly the PHQ-9 and HADS-D scores may be interpreted according to severity cut-offs. Table 3 shows the score distributions for these measures, revealing the lack of concurrence between the severity ranges; the PHQ-9 ‘mild depression’ category is particularly problematic as the recommended score range (5-9) falls below this measure’s standard cut-point for the presence of depression (≥10). Although scores on both the PHQ-9 and HADS-D assigned greater numbers of people to mild and moderate depression than the CIS-R standard, this tendency was more pronounced for the PHQ-9 which categorised around four times as many participants in this way than the CIS-R, whereas around mild and moderate depression was indicated likely in around three times as many patients by HADS-D score compared to the reference standard. 

**Table 3:** Distribution of participants by CIS-R, PHQ-9 and HADS-D severity ratings.

**CIS-R**	n (%)		**PHQ-9**	n (%)		**HADS-D**	n (%)	
No depression	702 (95.5)		No depression	196 (26.7)	634 (86.4)	No depression	653 (89.3)	
			Minimal depression (1-4)	296 (40.3)				
			Mild depression (5-9)	142 (19.4)				
Mild depression	13 (1.8)	33 (4.5)	Moderate depression (10-14)	61 (8.3)	100 (13.6)	Possible/ mild depression (8-10)	50 (6.8)	103 (10.6)
Moderate depression	9 (1.2)		Moderately severe depression (15-19)	27 (3.7)		Probable depression, moderate (≥11-15)	24 (3.3)	
Severe depression	11 (1.5)		Severe depression (≥20)	12 (1.6)		Probable depression, severe (>15)	4 (0.6)	

## Discussion

The main aim of this study was to determine the performance characteristics of two commonly used self-report case identification instruments, the PHQ-9 and the HADS-D, for depression relative to a referent diagnostic standard in primary care patients with CHD. The findings reveal high levels of internal consistency and substantial intercorrelations between both instruments which is indicative of their construct validity. 

Criterion validity for the PHQ-9 using both summed and categorical scoring was good. At the standard recommended cut-point of ≥10, findings within this primary care CHD sample were near identical to those derived from meta-analysis of six studies of this instrument’s use with patients recruited on the basis of their chronic medical conditions [23]. Whist the current study indicated sensitivity as 84% and specificity as 90%, the corresponding results of the meta-analysis were 84% and 88%. An improvement in PHQ-9 performance was evident upon application of a lower cut-point (of ≥8), resulting in increased sensitivity with only modest reduction in specificity. The PHQ-9 scored by diagnostic algorithm methods for major depression performed less well, though results were markedly better than in other studies with CHD patients [22,26], and were generally similar to those obtained from meta-analyses of PHQ-9 accuracy among patients in primary care and medical settings [41]. 

The performance of the HADS-D at the standard cut-point (of ≥8) was weaker, with a large proportion of true cases likely to be missed (sensitivity=53% specificity=91%). This result is similar to the findings from an Australian sample of patients 3-months following hospital discharge for CHD related events/procedures where a sensitivity of 46% and specificity of 92% were identified using this cut-point [22]. A meta-analysis of 29 studies using the HADS-D at standard cut-point provided a higher sensitivity 75%, with specificity 81% [22]. In the current study satisfactory performance of the HADS-D scale required a cut-point of ≥5, and though adequate, the resulting test characteristics were considerably weaker than the PHQ-9.

Other studies have identified the diagnostic superiority of the PHQ-9 over the HADS-D among patients with medical co-morbidities [24]. However, part of the reason for this is likely to be because many of these studies have used DSM-IV-based diagnostic measures as the criterion standard, and the PHQ-9 was developed to match DSM-IV criteria on an item-by-item basis, whereas the HADS-D was developed to assess depression in medically ill patients and its items are centred on loss of interest and pleasure with somatic features excluded from measurement. Hence, findings of high criterion validity for the PHQ-9 in relation to DSM-IV major depression relate in part to its content validity. A strength of the current study is that it differs from the majority of other evaluations of the PHQ-9 in that the criterion standard is based on ICD-10 criteria, so avoiding this element of circularity which may occur in psychometric evaluations of the PHQ-9 in relation to the diagnostic measure on which it is based. 

Another important strength of this study is its setting in a primary care population which is where most people with CHD are provided with ongoing monitoring and support, and hence the setting in which the opportunity and imperative for detecting depression is most clear. 

In line with expert recommendations we excluded from diagnostic accuracy analyses those patients that were either recognised or treated for depression at the time of the study. This approach is designed to ensure the avoidance of spectrum bias and associated overestimation of the accuracy of test measures, and in particular elevated values for the positive predictive value of the index tests. Although there was relatively little change in the sensitivity and specificity of the index measures associated with this procedure, the changed prevalence of depression in the sample population resulted in positive predictive values that were markedly reduced (at the optimal cut-points: PHQ-9 21% v 27%; PHQ-9 algorithm 37% v 46% ; HADS-D 14% v 20%).

The discrepant findings in relation to severity bandings between the PHQ-9 and HADS-D have been identified in previous primary care studies [20,29]. In part this may relate to the severity categories recommended by the PHQ-9’s authors: problematically the ‘mild depression’ banding does not include sufficient symptoms of adequate severity to meet major depression criteria of any severity, and falls below the standard cut-point for depressive episode/major depression; whilst the additional sub-division of ‘moderately severe’ depression does not link to diagnostic manuals and clinical guidelines. Interestingly, this categorisation of the PHQ-9 score is not adopted by the McArthur Foundation [42] initiative on depression and primary care (which notes PHQ-9 scores of 10-14 as indicative mild depression), nor was it used in a recent large-scale evaluation of the PHQ-9 in primary care [43]. 

Other studies have identified higher rates of depression among patients with CHD (and other chronic medical conditions) than our study: Rudisch and Nemeroff [44] reported prevalence rates for depression in CHD ranging from 17% to 27%, whilst post-MI prevalence rates have been noted to range between 16% and 27% [45]. It is possible that the pooled prevalence estimate of 20% reported for depression following MI as identified by diagnostic measure [45] may be affected by inaccuracies in determining depression rates in the largest of the studies included [46]. A lower prevalence of depression has been identified in community-based studies using a nationally representative population sample rather than one drawn from outpatients or people recently hospitalised: a study based on National Health Interview Survey data of 30 801 adults found the 12-month prevalence of major depression to be 9.3% in individuals with CHD as compared with 4.8% in those with no comorbid medical illness [47]. In general, a two- to three- fold increase in depression prevalence compared to the general population is observed, and in this study the prevalence of 7% indicated from CIS-R results may be compared with the British household population rate identified using the same measure of 2% for depressive episode [48]. 

A possible limitation of this study is that recruitment of the sample was limited to general practices based in South London, and only 28% of eligible patients participated in this study. This low participation rate relates in part to the recruitment strategy demanded by UK research ethics committees for primary care research which requires GPs to gain initial patient consent prior to researchers contacting patients to obtain fully informed consent. Comparison of the characteristics of the study sample with relevant data for England indicates generalizability. The study participants were predominantly (70%) male, whilst data for England indicate that 62% of CHD cases are male; the mean age of participants was 71 years, and data for England indicates that 93% of people with CHD are aged over 55 years and 79% aged over 65 years [49]. 2.1% of patients within the 16 included practices were on the QOF CHD registers, which compares with 2.2% for the London Strategic Health Authority area, 2.9% for the wider South Central area, and 3.5% for England (http://www.gpcontract.co.uk/child/ENG/CHD%201/11). The number of practices involved (n=16) together with the ethnic and socio-economic diversity provided by their differing geographical settings provide some indication that the participants reflect the spectrum of patients who would normally be seen in clinical practice, and that the results will generalise to the wider primary care population. 

## Conclusions

Despite these limitations, this study provides robust findings based on a large scale primary care population of people with CHD, showing the psychometric properties of commonly used depression case finding measures. The results indicate the superiority of the PHQ-9 for depression case identification in this patient group, and are largely consistent with the findings of reviews of depression identification tool performance among people with chronic physical health problems in showing acceptable diagnostic properties for this instrument. To our knowledge, this is the first study to compare the PHQ-9 and HADS-D against a diagnostic standard among patients with CHD in primary care; other similar studies have been based on patients from cardiology outpatient clinics, or following cardiac surgery, or post-myocardial infarction. Among this primary care CHD population the sensitivity (94%) and specificity (84%) of the PHQ-9 using a cut-point of at ≥8 were found to be better than the median values identified in a systematic review of screening instruments for depression in cardiovascular care (84% and 79% respectively) [25]. However, if the low depression prevalence (6.7% overall, but 4.4% not otherwise recognised) found in our study is accurate, this indicates that only around 20% of PHQ-9 newly identified cases are true cases; whilst if depression prevalence in this population were 20% [45], the true positive proportion would be around 60% for this instrument. With a depression prevalence of 12% (based on re-examination of ENRICHD data combined with other studies of post-MI patients), around 40% of identified cases would be true positives.

We know that depression is predictive of increased disability and raised mortality in people with CHD and that standard pharmacological and psychosocial interventions are effective in treating such co-morbid depression – not only for mental health outcomes, but emerging evidence indicates for cardiovascular outcomes and survival rates too [50]. Among CHD patients, the deleterious effects of comorbid depression coupled with its under-recognition indicate that accurate detection must remain a clinical priority. The findings of this study extend our knowledge of the characteristics of case identification measures to assist in this important area, and provide useful guidance on appropriate tools and optimal cut-off scores for use in primary care. However, the current study is not able to shed light on whether using case identification measures among this at-risk population is associated with increased delivery of appropriate clinical interventions to screen-positive patients or of real-world clinical outcome benefits. Recent retrospective examination of a large database of primary care patients in Scotland [51] indicated that screening those with either CHD or diabetes was associated a significant but small increase in new diagnoses of depression and a new course of antidepressant in the month after screening. However, these authors caution that the resource implications of such screening approaches may not be justified by the modest improvements beyond standard care. Although we have found the PHQ-9 to possess adequate diagnostic accuracy for this patient group, this finding is insufficient to determine whether using this instrument will result in improvement in patient management or outcomes.

## Supporting Information

Table S1 **Depressive disorder: PHQ-9 operating characteristics (complete).**
(DOCX)

Table S2 **Depressive disorder: PHQ-9 algorithm operating characteristics (complete).**
(DOCX)

Table S3 **Depressive disorder: HADS (D) operating characteristics (complete).**
(DOCX)

## References

[1] Kessler RC, Chiu WT, Demler O, Merikangas KR, Walters EE (2005) Prevalence, severity, and comorbidity of 12-month DSM-IV disorders in the national comorbidity survey replication. Arch Gen Psychiatry 62: 617-627. doi:10.1001/archpsyc.62.6.617. PubMed: 15939839. 15939839 PMC2847357

[2] Waraich P, Goldner EM, Somers JM, Hsu L (2004) Prevalence and incidence studies of mood disorders: A systematic review of the literature. Can J Psychiatry 49: 124-138. PubMed: 15065747. 15065747 10.1177/070674370404900208

[3] World Health Organization (2008) The global burden of disease: 2004 update. Geneva: WHO.

[4] Egede LE (2007) Failure to recognize depression in primary care: Issues and challenges. J Gen Intern Med 22: 701-703. doi:10.1007/s11606-007-0170-z. PubMed: 17370030. 17370030 PMC1852925

[5] Scott KM, Hwang I, Chiu WT, Kessler RC, Sampson NA et al. (2010) Chronic physical conditions and their association with first onset of suicidal behavior in the world mental health surveys. Psychosom Med 72: 712-719. doi:10.1097/PSY.0b013e3181e3333d. PubMed: 20498290. 20498290 PMC12980531

[6] Moussavi S, Chatterji S, Verdes E, Tandon A, Patel V et al. (2007) Depression, chronic diseases, and decrements in health: Results from the world health surveys. Lancet 370: 851-858. doi:10.1016/S0140-6736(07)61415-9. PubMed: 17826170. 17826170

[7] Lichtman JH, Bigger JT Jr, Blumenthal JA, Frasure-Smith N, Kaufmann PG et al. (2008) Depression and coronary heart disease: Recommendations for screening, referral, and treatment: A science advisory from the American Heart Association Prevention Committee of the Council on Cardiovascular Nursing, Council on Clinical Cardiology, Council on Epidemiology and Prevention, and Interdisciplinary Council on Quality of Care and Outcomes Research: Endorsed by the American Psychiatric Association. Circulation 118: 1768-1775. doi:10.1161/CIRCULATIONAHA.108.190769. PubMed: 18824640. 18824640

[8] Barth J, Schumacher M, Herrmann-Lingen C (2004) Depression as a risk factor for mortality in patients with coronary heart disease: A meta-analysis. Psychosom Med 66: 802-813. doi:10.1097/01.psy.0000146332.53619.b2. PubMed: 15564343. 15564343

[9] Mitchell AJ, Vaze A, Rao S (2009) Clinical diagnosis of depression in primary care: A meta-analysis. Lancet 374: 609-619. doi:10.1016/S0140-6736(09)60879-5. PubMed: 19640579. 19640579

[10] Rost K, Nutting P, Smith J, Coyne JC, Cooper-Patrick L et al. (2000) The role of competing demands in the treatment provided primary care patients with major depression. Arch Fam Med 9: 150-154. doi:10.1001/archfami.9.2.150. PubMed: 10693732. 10693732

[11] Menchetti M, Belvederi Murri MB, Bertakis K, Bortolotti B, Berardi D (2009) Recognition and treatment of depression in primary care: Effect of patients' presentation and frequency of consultation. J Psychosom Res 66: 335-341. doi:10.1016/j.jpsychores.2008.10.008. PubMed: 19302892. 19302892

[12] U.S. Preventive Services Task Force (2009) Screening for depression in adults: Recommendation statemen t. http://www.uspreventiveservicestaskforce.org/uspstf09/adultdepression/addeprrs.htm 10.7326/0003-4819-151-11-200912010-00002 19949139

[13] Gilbody S, Sheldon T, House A (2008) Screening and case-finding instruments for depression: A meta-analysis. CMAJ 178: 997-1003. doi:10.1503/cmaj.070281. PubMed: 18390942. 18390942 PMC2276549

[14] NICE (2009) Depression in adults with a chronic physical health problem: Treatment and management (national clinical practice guideline 91). London: National Institute for Health and Clinical Excellence.

[15] NICE (2009) Depression in adults (update). Depression: The treatment and management of depression in adults (national clinical practice guideline 90). London: The British Psychological Society and the Royal College of Psychiatrists (commissioned by: National Institute for Health and Clinical Excellence).

[16] Whooley MA, Avins AL, Miranda J, Browner WS (1997) Case-finding instruments for depression. Two questions are as good as many. J Gen Intern Med 12: 439-445. doi:10.1046/j.1525-1497.1997.00076.x. PubMed: 9229283. 9229283 PMC1497134

[17] Hasnain M, Vieweg WV, Lesnefsky EJ, Pandurangi AK (2011) Depression screening in patients with coronary heart disease: A critical evaluation of the AHA guidelines. J Psychosom Res 71: 6-12. doi:10.1016/j.jpsychores.2010.10.009. PubMed: 21665006. 21665006

[18] Thombs BD, Roseman M, Coyne JC, de Jonge P, Delisle VC et al. (2013) Does evidence support the american heart association's recommendation to screen patients for depression in cardiovascular care? An updated systematic review. PLOS ONE 8: e52654. doi:10.1371/journal.pone.0052654. PubMed: 23308116. 23308116 PMC3538724

[19] Thombs BD, Coyne JC, Cuijpers P, de Jonge P, Gilbody S et al. (2012) Rethinking recommendations for screening for depression in primary care. CMAJ 184: 413-418. doi:10.1503/cmaj.109-4183. PubMed: 21930744. 21930744 PMC3291670

[20] Cameron IM, Crawford JR, Lawton K, Reid IC (2008) Psychometric comparison of PHQ-9 and HADS for measuring depression severity in primary care. Br J Gen Pract 58: 32-36. doi:10.3399/bjgp08X263794. PubMed: 18186994. 18186994 PMC2148236

[21] Löwe B, Spitzer RL, Gräfe K, Kroenke K, Quenter A et al. (2004) Comparative validity of three screening questionnaires for DSM-IV depressive disorders and physicians' diagnoses. J Affect Disord 78: 131-140. doi:10.1016/S0165-0327(02)00237-9. PubMed: 14706723. 14706723

[22] Stafford L, Berk M, Jackson HJ (2007) Validity of the Hospital Anxiety and Depression Scale and Patient Health Questionnaire-9 to screen for depression in patients with coronary artery disease. Gen Hosp Psychiatry 29: 417-424. doi:10.1016/j.genhosppsych.2007.06.005. PubMed: 17888808. 17888808

[23] McManus D, Pipkin SS, Whooley MA (2005) Screening for depression in patients with coronary heart disease (data from the Heart and Soul study). Am J Cardiol 96: 1076-1081. doi:10.1016/j.amjcard.2005.06.037. PubMed: 16214441. 16214441 PMC2776683

[24] Meader N, Mitchell AJ, Chew-Graham C, Goldberg D, Rizzo M et al. (2011) Case identification of depression in patients with chronic physical health problems: A diagnostic accuracy meta-analysis of 113 studies. Br J Gen Pract 61: 808-820. doi:10.3399/bjgp11X613151. PubMed: 22137418. PMC3223779 22137418

[25] Thombs BD, de Jonge P, Coyne JC, Whooley MA, Frasure-Smith N et al. (2008) Depression screening and patient outcomes in cardiovascular care: A systematic review. JAMA 300: 2161-2171. doi:10.1001/jama.2008.667. PubMed: 19001627. 19001627

[26] Thombs BD, Ziegelstein RC, Whooley MA (2008) Optimizing detection of major depression among patients with coronary artery disease using the Patient Health Questionnaire: Data from the Heart and Soul study. J Gen Intern Med 23: 2014-2017. doi:10.1007/s11606-008-0802-y. PubMed: 18815842. 18815842 PMC2596499

[27] Strik JJ, Honig A, Lousberg R, Denollet J (2001) Sensitivity and specificity of observer and self-report questionnaires in major and minor depression following myocardial infarction. Psychosomatics 42: 423-428. doi:10.1176/appi.psy.42.5.423. PubMed: 11739910. 11739910

[28] Thombs BD, Arthurs E, El-Baalbaki G, Meijer A, Ziegelstein RC et al. (2011) Risk of bias from inclusion of patients who already have diagnosis of or are undergoing treatment for depression in diagnostic accuracy studies of screening tools for depression: Systematic review. BMJ 343: d4825. doi:10.1136/bmj.d4825. PubMed: 21852353. 21852353 PMC3191850

[29] Kendrick T, Dowrick C, McBride A, Howe A, Clarke P et al. (2009) Management of depression in UK general practice in relation to scores on depression severity questionnaires: Analysis of medical record data. BMJ 338: b750. doi:10.1136/bmj.b750. PubMed: 19299475. 19299475

[30] Tylee A, Ashworth M, Barley E, Brown J, Chambers J et al. (2011) Up-beat UK: A programme of research into the relationship between coronary heart disease and depression in primary care patients. BMC family practice 12: 38. doi:10.1186/1471-2296-12-38. PubMed: 21605435. 21605435 PMC3120657

[31] Spitzer RL, Kroenke K, Williams JB (1999) Validation and utility of a self-report version of PRIME-MD: The PHQ primary care study. JAMA 282: 1737-1744. doi:10.1001/jama.282.18.1737. PubMed: 10568646. 10568646

[32] American Psychiatric Association (2000) Diagnostic and Statistical Manual of Mental Disorders, 4th edn, text revision ( DSM IV-TR). Washington, DC: APA.

[33] Kroenke K, Spitzer RL, Williams JB (2001) The PHQ-9: Validity of a brief depression severity measure. J Gen Intern Med 16: 606-613. doi:10.1046/j.1525-1497.2001.016009606.x. PubMed: 11556941. 11556941 PMC1495268

[34] Zigmond AS, Snaith RP (1983) The Hospital Anxiety and Depression Scale. Acta Psychiatr Scand 67: 361-370. doi:10.1111/j.1600-0447.1983.tb09716.x. PubMed: 6880820. 6880820

[35] Olssøn I, Mykletun A, Dahl AA (2005) The Hospital Anxiety and Depression rating scale: A cross-sectional study of psychometrics and case finding abilities in general practice. BMC Psychiatry 5: 46-doi. doi:10.1186/1471-244X-5-46. PubMed: 16351733. 16351733 PMC1343544

[36] Bjelland I, Dahl AA, Haug TT, Neckelmann D (2002) The validity of the Hospital Anxiety and Depression Scale. An updated literature review. J Psychosom Res 52: 69-77. doi:10.1016/S0022-3999(01)00296-3. PubMed: 11832252. 11832252

[37] Barth J, Martin CR (2005) Factor structure of the Hospital Anxiety and Depression Scale (HADS) in German coronary heart disease patients. Health Qual Life Outcomes 3: 15. doi:10.1186/1477-7525-3-15. PubMed: 15771778. 15771778 PMC555847

[38] Wilkinson MJ, Barczak P (1988) Psychiatric screening in general practice: Comparison of the General Health Questionnaire and the Hospital Anxiety Depression Scale. J R Coll Gen Pract 38: 311-313. PubMed: 3255827. 3255827 PMC1711493

[39] Lewis G, Pelosi AJ, Araya R, Dunn G (1992) Measuring psychiatric disorder in the community: A standardized assessment for use by lay interviewers. Psychol Med 22: 465-486. doi:10.1017/S0033291700030415. PubMed: 1615114. 1615114

[40] Jordanova V, Wickramesinghe C, Gerada C, Prince M (2004) Validation of two survey diagnostic interviews among primary care attendees: A comparison of CIS-R and CIDI with SCAN ICD-10 diagnostic categories. Psychol Med 34: 1013-1024. doi:10.1017/S0033291703001727. PubMed: 15554572. 15554572

[41] Wittkampf K, van Ravesteijn H, Baas K, van de Hoogen HH, Schene A et al. (2009) The accuracy of Patient Health Questionnaire-9 in detecting depression and measuring depression severity in high-risk groups in primary care. Gen Hosp Psychiatry 31: 451-459. doi:10.1016/j.genhosppsych.2009.06.001. PubMed: 19703639. 19703639

[42] MacArthur Foundation (2004) The MacArthur initiative on depression and primary care. Available: http://www.depression-primarycare.org.2012.

[43] Arroll B, Goodyear-Smith F, Crengle S, Gunn J, Kerse N et al. (2010) Validation of PHQ-2 and PHQ-9 to screen for major depression in the primary care population. Ann Fam Med 8: 348-353. doi:10.1370/afm.1139. PubMed: 20644190. 20644190 PMC2906530

[44] Rudisch B, Nemeroff CB (2003) Epidemiology of comorbid coronary artery disease and depression. Biol Psychiatry 54: 227-240. doi:10.1016/S0006-3223(03)00587-0. PubMed: 12893099. 12893099

[45] Thombs BD, Bass EB, Ford DE, Stewart KJ, Tsilidis KK et al. (2006) Prevalence of depression in survivors of acute myocardial infarction. J Gen Intern Med 21: 30-38. doi:10.1007/s11606-006-0272-z. PubMed: 16423120. 16423120 PMC1484630

[46] ENRICHD Investigators (2001) Enhancing recovery in coronary heart disease (ENRICHD): Baseline characteristics. Am J Cardiol 88: 316-322. doi:10.1016/S0002-9149(01)01652-6. PubMed: 11472719. 11472719

[47] Egede LE (2007) Major depression in individuals with chronic medical disorders: Prevalence, correlates and association with health resource utilization, lost productivity and functional disability. Gen Hosp Psychiatry 29: 409-416. doi:10.1016/j.genhosppsych.2007.06.002. PubMed: 17888807. 17888807

[48] McManus S, Meltzer H, Brugha T, Bebbington P, Jenkins R (2009) Adult psychiatric morbidity in England, 2007: Results of a household survey. Leeds: The NHS Information Centre for Health and Social Care..

[49] Townsend N, Wickramasinghe K, Bhatnagar P, Smolina K, Nichols M et al. (2012) Coronary heart disease statistics: A compendium of health statistics, 2012 edition.

[50] Rutledge T, Redwine LS, Linke SE, Mills PJ (2013) A meta-analysis of mental health treatments and cardiac rehabilitation for improving clinical outcomes and depression among patients with coronary heart disease. Psychosom Med 75: 335-349. doi:10.1097/PSY.0b013e318291d798. PubMed: 23630306. 23630306

[51] Burton C, Simpson C, Anderson N (2013) Diagnosis and treatment of depression following routine screening in patients with coronary heart disease or diabetes: A database cohort study. Psychol Med 43: 529-537. doi:10.1017/S0033291712001481. PubMed: 22804849. 22804849

