# Supplementary material for: Detecting Depression in Patients with Coronary Heart Disease: a Diagnostic Evaluation of the PHQ-9 and HADS-D in Primary Care, Findings From the UPBEAT-UK Study
Source: PLoS One. 2013 Oct 10;8(10):e78493. doi: 10.1371/journal.pone.0078493 (PMC3795055; doi:10.1371/journal.pone.0078493)
Supplement: Table S1 — Depressive disorder: PHQ-9 operating characteristics (complete). (DOCX) [file pone.0078493.s001.docx]

Table S1: Depressive disorder: PHQ-9 operating characteristics (complete)

|  | Sensitivity (95%CI) | Specificity (95% CI) | Positive LikelihoodRatio | Negative Likelihood ratio | Youden Index | Positive Predictive Value (%) | Negative Predictive Value (%) |
| --- | --- | --- | --- | --- | --- | --- | --- |
| **PHQ (n=730)** |  |  |  |  |  |  |  |
| Cut-off point ≥0 | 100.0 (89.1, 100.0) | 0.0 (0.0, 0.5) | 1.0 | / | 0.00 | 4.4 | / |
| Cut-off point ≥1 | 100.0 (89.1, 100.0) | 28.1 (24.8, 31.6) | 1.4 | 0.0 | 0.28 | 6.0 | 100.0 |
| Cut-off point ≥2 | 100.0 (89.1, 100.0) | 42.4 (38.7, 46.2) | 1.7 | 0.0 | 0.42 | 7.4 | 100.0 |
| Cut-off point ≥3 | 100.0 (89.1, 100.0) | 53.2 (49.4, 56.9) | 2.1 | 0.0 | 0.53 | 8.9 | 100.0 |
| Cut-off point ≥4 | 100.0 (89.1, 100.0) | 62.6 (58.9, 66.2) | 2.7 | 0.0 | 0.63 | 10.9 | 100.0 |
| Cut-off point ≥5 | 100.0 (89.1, 100.0) | 70.5 (67.0, 73.8) | 3.4 | 0.0 | 0.70 | 13.5 | 100.0 |
| Cut-off point ≥6 | 100.0 (89.1, 100.0) | 76.4 (73.0, 79.5) | 4.2 | 0.0 | 0.76 | 16.2 | 100.0 |
| Cut-off point ≥7 | 93.8 (79.2, 99.2) | 80.7 (77.5, 83.5) | 4.8 | 0.1 | 0.74 | 18.2 | 99.7 |
| Cut-off point ≥8 | 93.8 (79.2, 99.2) | 83.8 (80.9, 86.5) | 5.8 | 0.1 | 0.78 | 21.0 | 99.7 |
| Cut-off point ≥9 | 87.5 (71.0, 96.5) | 86.7 (83.9, 89.1) | 6.6 | 0.1 | 0.74 | 23.1 | 99.3 |
| Cut-off point ≥10 | 84.4 (67.2, 94.7) | 89.8 (87.3, 92.0) | 8.3 | 0.2 | 0.74 | 27.6 | 99.2 |
| Cut-off point ≥11 | 81.3 (63.6, 92.8) | 91.4 (89.1, 93.4) | 9.5 | 0.2 | 0.73 | 30.2 | 99.1 |
| Cut-off point ≥12 | 75.0 (56.6, 88.5) | 93.8 (91.8, 95.5) | 12.2 | 0.3 | 0.69 | 35.8 | 98.8 |
| Cut-off point ≥13 | 56.3 (37.7, 73.6) | 95.3 (93.4, 96.7) | 11.9 | 0.5 | 0.52 | 35.3 | 97.9 |
| Cut-off point ≥14 | 53.1 (34.7, 70.9) | 96.3 (94.6, 97.6) | 14.3 | 0.5 | 0.49 | 39.5 | 97.8 |
| Cut-off point ≥15 | 53.1 (34.7, 70.9) | 97.0 (95.4, 98.1) | 17.7 | 0.5 | 0.50 | 44.7 | 97.8 |
| Cut-off point ≥16 | 37.5 (21.1, 56.3) | 97.7 (96.3, 98.7) | 16.4 | 0.6 | 0.35 | 42.9 | 97.2 |
| Cut-off point ≥17 | 31.3 (16.1, 50.0) | 98.1 (96.8, 99.0) | 16.8 | 0.7 | 0.29 | 43.5 | 96.9 |
| Cut-off point ≥18 | 28.1 (13.7, 46.7) | 98.6 (11.4, 16.7) | 19.6 | 0.7 | 0.27 | 47.4 | 96.8 |
| Cut-off point ≥19 | 25.0 (11.5, 43.4) | 99.0 (97.9, 99.6) | 24.9 | 0.8 | 0.24 | 53.3 | 96.6 |
| Cut-off point ≥20 | 25.0 (11.5, 43.4) | 99.4 (98.5, 99.8) | 43.6 | 0.8 | 0.24 | 66.7 | 96.7 |
| Cut-off point ≥21 | 21.9 (9.3, 40.0) | 99.7 (99.0, 100.0) | 76.3 | 0.8 | 0.22 | 77.8 | 96.5 |
| Cut-off point ≥22 | 15.6 (5.3, 32.8) | 99.9 (99.2, 100.0) | 109.1 | 0.8 | 0.15 | 83.3 | 96.2 |
| Cut-off point ≥23 | 12.5 (3.5, 29.0) | 99.9 (99.2, 100.0) | 87.3 | 0.9 | 0.12 | 80.0 | 96.1 |
| Cut-off point ≥24 | 9.4 (2.0, 25.0) | 99.9 (99.2, 100.0) | 65.4 | 0.9 | 0.09 | 75.0 | 96.0 |
| Cut-off point >24 | 0.0 (0.0, 10.9) | 100.0 (99.5, 100.0) | / | 1.0 | 0.00 | / | 95.6 |
