# Supplementary material for: Detecting Depression in Patients with Coronary Heart Disease: a Diagnostic Evaluation of the PHQ-9 and HADS-D in Primary Care, Findings From the UPBEAT-UK Study
Source: PLoS One. 2013 Oct 10;8(10):e78493. doi: 10.1371/journal.pone.0078493 (PMC3795055; doi:10.1371/journal.pone.0078493)
Supplement: Table S2 — Depressive disorder: PHQ-9 algorithm operating characteristics (complete). (DOCX) [file pone.0078493.s002.docx]

Table S2: Depressive disorder: PHQ-9 algorithm operating characteristics (complete)

|  | Sensitivity (95%CI) | Specificity (95% CI) | Positive Likelihood Ratio | Negative Likelihood ratio | Youden Index | Positive Predictive Value (%) | Negative Predictive Value (%) |
| --- | --- | --- | --- | --- | --- | --- | --- |
| **PHQ-9 Categorical algorithm (n=730)** |  |  |  |  |  |  |  |
| Cut-off point ≥0 (no depression) | 100.0 (89.1, 100.0) | 0.0 (0.0, 0.5) | 1.0 | / | 0.000 | 4.4 | / |
| Cut-off point ≥1 (other depression) | 93.8 (79.2, 99.2) | 83.5 (80.6, 86.2) | 5.7 | 0.1 | 0.773 | 20.7 | 99.7 |
| Cut-off point ≥2 (major depression | 59.4 (40.6, 76.3) | 95.3 (93.4, 96.7) | 12.6 | 0.4 | 0.547 | 36.5 | 98.1 |
| Cut-off point >2 (major depression | 0.0 (0.0, 10.9) | 100.0 (99.5, 100.0) | / | 1.0 | 0.000 | / | 95.6 |
