# Supplementary material for: Detecting Depression in Patients with Coronary Heart Disease: a Diagnostic Evaluation of the PHQ-9 and HADS-D in Primary Care, Findings From the UPBEAT-UK Study
Source: PLoS One. 2013 Oct 10;8(10):e78493. doi: 10.1371/journal.pone.0078493 (PMC3795055; doi:10.1371/journal.pone.0078493)
Supplement: Table S3 — Depressive disorder: HADS (D) operating characteristics (complete). (DOCX) [file pone.0078493.s003.docx]

Table S3: Depressive disorder: HADS (D) operating characteristics (complete)

|  | Sensitivity (95%CI) | Specificity (95% CI) | Positive Likelihood Ratio | Negative Likelihood ratio | Youden Index | Positive Predictive Value (%) | Negative Predictive Value (%) |
| --- | --- | --- | --- | --- | --- | --- | --- |
| **HADS(n=730)** |  |  |  |  |  |  |  |
| Cut-off point ≥0 | 100.0 (89.1, 100.0) | 0.0 (0.0, 0.5) | 1.0 | / | 0.00 | 4.4 | / |
| Cut-off point ≥1 | 100.0 (89.1, 100.0) | 23.2 (20.1, 26.5) | 1.3 | 0.0 | 0.23 | 5.6 | 100.0 |
| Cut-off point ≥2 | 100.0 (89.1, 100.0) | 45.1 (41.4, 48.9) | 1.8 | 0.0 | 0.45 | 7.7 | 100.0 |
| Cut-off point ≥3 | 100.0 (79.2, 99.2) | 57.0 (53.3, 60.7) | 2.3 | 0.0 | 0.57 | 9.6 | 100.0 |
| Cut-off point ≥4 | 93.8 (63.6, 92.8) | 67.8 (64.2, 71.2) | 2.9 | 0.1 | 0.62 | 11.8 | 99.6 |
| Cut-off point ≥5 | 81.3 (46.8, 81.4) | 76.7 (73.3, 79.7) | 3.5 | 0.2 | 0.58 | 13.8 | 98.9 |
| Cut-off point ≥6 | 65.6 (40.6, 76.3) | 83.1 (80.1, 85.8) | 3.9 | 0.4 | 0.49 | 15.1 | 98.1 |
| Cut-off point ≥7 | 59.4 (34.7, 70.9) | 88.3 (85.6, 90.5) | 5.1 | 0.5 | 0.48 | 18.8 | 97.9 |
| Cut-off point ≥8 | 53.1 (26.4, 62.3) | 91.4 (89.1, 93.4) | 6.2 | 0.5 | 0.45 | 22.1 | 97.7 |
| Cut-off point ≥9 | 43.8 (23.7, 59.4) | 94.6 (92.6, 96.1) | 8.0 | 0.6 | 0.38 | 26.9 | 97.4 |
| Cut-off point ≥10 | 40.6 (18.6, 53.2) | 96.3 (94.6, 97.6) | 10.9 | 0.6 | 0.37 | 33.3 | 97.3 |
| Cut-off point ≥11 | 34.4 (7.2, 36.4) | 97.7 (96.3, 98.7) | 15.0 | 0.7 | 0.32 | 40.7 | 97.0 |
| Cut-off point ≥12 | 18.8 (2.0, 25.0) | 98.6 (97.4, 99.3) | 13.1 | 0.8 | 0.17 | 37.5 | 96.4 |
| Cut-off point ≥13 | 9.4 (0.8, 20.8) | 99.3 (98.3, 99.8) | 13.1 | 0.9 | 0.09 | 37.5 | 96.0 |
| Cut-off point ≥14 | 6.3 (0.0, 10.9) | 99.4 (98.5, 99.8) | 10.9 | 0.9 | 0.06 | 33.3 | 95.9 |
| Cut-off point ≥16 | 0.0 (0.0, 10.9) | 99.4 (98.5, 99.8) | 0.0 | 1.0 | -0.01 | 0.0 | 95.6 |
| Cut-off point ≥17 | 0.0 (0.0, 10.9) | 99.6 (98.7, 99.9) | 0.0 | 1.0 | 0.00 | 0.0 | 95.6 |
| Cut-off point ≥18 | 0.0 (0.0, 10.9) | 99.7 (99.0, 100.0) | 0.0 | 1.0 | 0.00 | 0.0 | 95.6 |
| Cut-off point >18 | 0.0 (0.0, 10.9) | 100.0 (99.5, 100.0) | / | 1.0 | 0.00 | / | 95.6 |
| Note: Cut-off point ≥15 on HADS missing as no participant scored this | | | | | | | |
